# Dendritic cell vaccination in combination with erlotinib in a patient with inoperable lung adenocarcinoma: a case report

**DOI:** 10.1186/s13256-024-04363-z

**Published:** 2024-02-10

**Authors:** Takuya Kosumi, Masanori Kobayashi, Shigetaka Shimodaira, Haruo Sugiyama, Shigeo Koido

**Affiliations:** 1 Kyushukouseikai Clinic, 1-2-12 Tenjin, Chuo-Ku, Fukuoka-Shi, 810-0001 Japan; 2 Okazakiyuuai Clinic, 104-1 Azaikeda, Tsutsubaricho, Okazaki-Shi, Aichi-ken 444-0932 Japan; 3 Department of Regenerative Medicine, Kanazawa Medical University, Kahoku, Ishikawa 920-0293 Japan; 4 Department of Cancer Immunology, Osaka University Graduate School of Medicine, Suita-City, Osaka 565-0871 Japan; 5 Division of Gastroenterology and Hepatology, Department of Internal Medicine, Kashiwa Hospital, The Jikei University School of Medicine, 163-1 Kashiwa-Shita, Kashiwa, Chiba 277-8567 Japan

**Keywords:** Cancer vaccines, Dendritic cells, WT1, MUC1, Lung cancer

## Abstract

**Background:**

Satisfactory treatment for patients with unresectable advanced lung cancer has not yet been established. We report a case of unresectable advanced lung cancer (stage IIIb: T2aN3M0) treated with a total of 15 doses of dendritic cells pulsed with a Wilms’ tumor 1 and mucin 1 vaccine in combination with erlotinib, a small molecule epidermal growth factor receptor tyrosine kinase inhibitor, for more than 699 days without recurrence or metastasis.

**Case presentation:**

A 63-year-old Korean woman was diagnosed with lung adenocarcinoma by pathology and computed tomography. The adenocarcinoma showed an epidermal growth factor receptor (EGFR) mutation, no anaplastic lymphoma kinase expression, and less than 1% expression of programmed death ligand 1. She received erlotinib alone for approximately 1 month. She then received erlotinib and the dendritic cells pulsed with Wilms’ tumor 1 and mucin 1 vaccine. The diameter of the erythema at the vaccinated sites was 30 mm at 48 hours after the first vaccination. Moreover, it was maintained at more than 20 mm during the periods of vaccination. These results suggested the induction of antitumor immunity by the vaccine. Remarkably, the tumor size decreased significantly to 12 mm, a 65.7% reduction, after combined therapy with eight doses of the dendritic cells pulsed with Wilms’ tumor 1 and mucin 1 vaccine and erlotinib for 237 days based on fluorodeoxyglucose uptake by positron emission tomography/computed tomography and computed tomography. Interestingly, after 321 days of combination therapy, the clinical findings improved, and no tumor was detected based on computed tomography. Validation of the tumor’s disappearance persisted for at least 587 days after treatment initiation, without any indication of recurrence or metastasis.

**Conclusion:**

Standard anticancer therapy combined with the dendritic cells pulsed with Wilms’ tumor 1 and mucin 1 vaccine may have therapeutic effects for such patients with unresectable lung adenocarcinoma.

## Background

Lung cancer is currently the leading cause of cancer-related death worldwide. Patients with non-small cell lung cancer (NSCLC) typically have a significantly poorer prognosis, despite major advances in the detection, treatment, and management of this deadly disease [1]. While immune checkpoint inhibitors such as ipilimumab and nivolumab have recently been shown to prolong overall survival (OS) and progression-free survival (PFS), only a minority of patients benefit clinically from these treatments, suggesting that in patients with NSCLC, the immune system has not yet fully realized its antitumor potential [2]. In patients with advanced NSCLC, the immunosuppressive milieu of the tumor microenvironment (TME) limits the induction of the patient’s antitumor immune response [3]. Therefore, novel therapeutic strategies to enhance the immune system are urgently needed. Due to the essential role of dendritic cells (DCs) in the adaptive immune response and their extensive crosstalk with immune-related cells, DC vaccines may be one of the most promising cellular vaccination strategies for patients with NSCLC.

Dendritic cell (DC) vaccination strategies have been developed as a therapy to overcome immune suppression in the TME and promote antitumor immune responses [2]. DCs are specialized antigen-presenting cells (APCs) that play an important role in initiating and regulating antitumor T-cell responses [4]. Interactions between major histocompatibility complex (MHC) molecules on DCs and T-cell receptors on T cells in the context of costimulatory molecules are essential for the induction of antitumor immunity [3]. Cancer vaccines using DCs loaded with tumor-associated antigen (TAA)-derived peptides, tumor lysates, mRNA, DNA, or whole tumor cells have been clinically applied and shown to induce antitumor immunity in many patients with cancer [4]. In clinical trials, MHC class I-restricted peptide-loaded DCs have been most commonly used to induce antigen-specific CD8^+^ cytotoxic T lymphocyte (CTL) responses to eradicate tumors, including lung cancer [5].

The Wilms’ tumor gene WT1 encodes a transcription factor that plays an important role in cell growth and differentiation. The WT1 gene is overexpressed in several tumor types, including lung cancer [6]. Importantly, WT1 was at the top of a prioritization of 75 TAAs based on multiple criteria, including therapeutic function and immunogenicity; MUC1 was ranked second [7]. MUC1 is a cell membrane glycoprotein that is also overexpressed in NSCLC and has been implicated in the carcinogenesis of premalignant lung lesions; thus, MUC1 has been used as a target for cancer vaccine strategies [8]. Interestingly, WT1-expressing cancer stem cells are completely eradicated by the WT1 immune response, which is essential for cancer cure; hence, this uniqueness of WT1 greatly contributes to cancer cure [6]. Accumulating clinical results suggest that major advances, such as the incorporation of DCs pulsed with WT1 and/or MUC1 peptide vaccines and standard anticancer therapies into the clinician’s therapeutic arsenal, can prolong OS and/or PFS in many patients with cancer [5]. Importantly, a previous report indicated that DCs pulsed with WT1 and/or MUC1 peptide vaccine had a significant impact on prolonging OS in patients with advanced NSCLC [6]. Here, we report the case of a patient with lung cancer with advanced adenocarcinoma who achieved excellent therapeutic results with a combination of DC vaccination and erlotinib.

## Case presentation

A 63-year-old Korean woman was diagnosed with stage IIIb (T2aN3M0) left lower lobe lung cancer (35 mm × 30 mm) by computed tomography (CT) and fluorodeoxyglucose (FDG)-positron emission tomography/CT (PET/CT). A CT-guided lung biopsy revealed adenocarcinoma. The adenocarcinoma showed epidermal growth factor receptor (EGFR) mutation, no anaplastic lymphoma kinase (ALK) expression, and less than 1% expression of programmed death ligand 1 (PD-L1) by immunohistochemistry. All serum tumor markers analyzed, including carcinoembryonic antigen (CEA), carbohydrate antigen 19–9 (CA19-9), cancer antigen 125 (CA125), and cancer antigen 15–3 (CA15-3), were found to be within normal ranges prior to treatment. The patient had an Eastern Cooperative Oncology Group performance status (PS) of 0, normal organ function, and no prior chemotherapy.

**Fig. 1:**
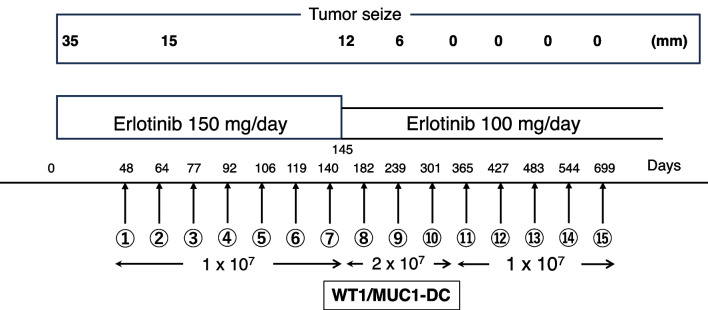
Treatment schedule and tumor size. The patient received erlotinib alone, a small molecule EGFR tyrosine kinase inhibitor, for approximately 1 month. The WT1/MUC1-DC vaccine suspension (approximately 1–2 × 10^7^ cells/dose) was administered intradermally to both upper arms at intervals of approximately 14–21 days independent of the erlotinib regimen. At the patient’s request, all 15 vaccine doses were administered following the completion of 699 days of treatment. After 321 days of combination therapy, no tumor was detected. Validation of the tumor’s disappearance persisted for at least 587 days after treatment initiation

**Fig. 2:**
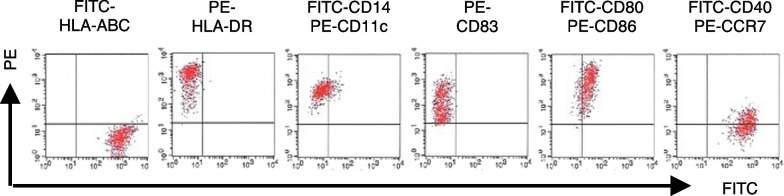
Phenotypic characterization of the WT1/MUC1-DC cells. The WT1/MUC1-DC cells were stained with the following monoclonal antibodies: fluorescein isothiocyanate (FITC)-conjugated anti HLA-ABC, anti CD14, anti CD80, anti CD40, and hycoerythrin (PE)-conjugated anti HLA-DR, anti CD11c, anti CD83, anti CD86, and anti CCR7. Phenotypic characterization of the WT1/MUC1-DC cells was analyzed by two-color flow cytometry for expression of HLA-ABC, HLA-DR, CD14, CD11c, CD83, CD80, CD86, CD40, and CCR7

The patient was initially treated with erlotinib (150 mg/day orally), a small molecule EGFR tyrosine kinase inhibitor, for approximately 1 month (Fig. 1). The patient then received erlotinib and DCs pulsed with MHC class II-restricted WT1 peptide and mucin 1 (MUC1) peptide vaccine (WT1/MUC1-DC) to induce WT1 and MUC1 antitumor CTLs. In the clinical setting, we did not include immunohistochemistry to select WT1 and MUC1 peptides, because previous studies have shown overexpression of WT1 [6] and MUC1 [8] in NSCLC. The WT1/MUC1-DC vaccine was prepared as previously reported [5, 9, 10]. Briefly, DCs were generated from peripheral blood mononuclear cells (PBMCs) prepared from leukapheresis products using Ficoll–Plaque Premium density gradient solution as previously described [11]. The patient’s human leukocyte antigen (HLA) types were HLA-A*(31:01/33:03), HLA-DRB1*(04:06/13:02), and HLA-DPB1*(02:01/04:01). Because WT1 killer peptides restricted to HLA-A*31:01/33:03 were not available, only class II binding helper peptides were used for WT1 vaccination. Therefore, the WT1 helper peptide (WT1 332–347; amino acid sequence, KRYFKLSHLQMHSRKH) was used for all MHC class II types [11]. The MUC1 long peptide (TRPAPGSTAPPAHGVTSAPDTRPAPGSTAP) was used for all MHC class I types [9]. The WT1/MUC1-DC vaccine was characterized by flow cytometry to ensure that it achieved the typical phenotype of professional APCs. The phenotype showed high levels of HLA-ABC, HLA-DR, CD11c, CD80, CD86, CD83, CD40, and CCR7 and low levels of CD14 (Fig. 2). WT1/MUC1-DCs were cryopreserved until the day of administration. The WT1/MUC1-DC vaccine suspension (approximately 1 × 10^7^ cells/dose) was diluted with saline to a total volume of 1.0 mL and administered intradermally to both upper arms at approximately 2–3 week intervals for up to seven vaccinations. Our protocol considered a course of seven vaccinations. After a single course of vaccination, more vaccines were available and vaccination intervals were longer. Therefore, from eight to ten vaccinations, double doses (approximately 2 × 10^7^ cells/dos) of WT1/MUC1-DC vaccine were administered at intervals of approximately 1–2 months. Patients then received the regular dose of WT1/MUC1-DC vaccine (approximately 1 × 10^7^ cells/dose) every 2 months from vaccinations 11 to 14 to maintain antitumor immunity. The last vaccination (approximately 1 × 10^7^ cells/dose) was administered 155 days after 14 vaccinations (699 days after treatment) (Fig. 1 and Table 1). The vaccine was administered independent of the erlotinib regimen.

**Table 1:** Schedule of WT1/MUC1-DC vaccine

Days after treatment	Vacciniations (Times)	Intervals of vaccinations (days)	Dose of vaccines (cell numbers)
0			
48	1		
64	2	16	1 × 10^7^
77	3	13	1 × 10^7^
92	4	15	1 × 10^7^
106	5	14	1 × 10^7^
119	6	13	1 × 10^7^
140	7	21	1 × 10^7^
182	8	42	2 × 10^7^
239	9	57	2 × 10^7^
301	10	62	2 × 10^7^
365	11	64	1 × 10^7^
427	12	62	1 × 10^7^
483	13	56	1 × 10^7^
544	14	61	1 × 10^7^
699	15	155	1 × 10^7^

**Fig. 3:**
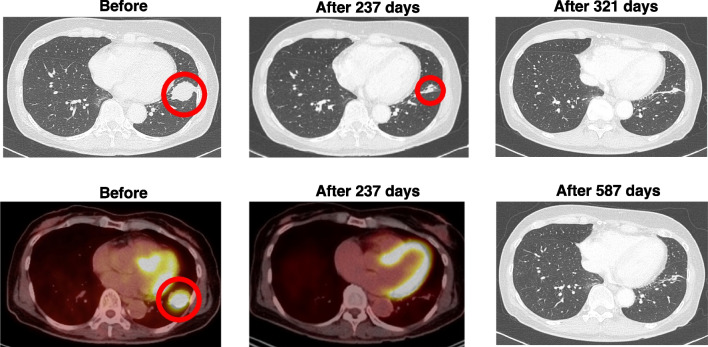
Lung cancer assessed by CT and PET/CT. The patient was diagnosed with lung cancer (35 mm × 30 mm) by computed tomography (CT) and fluorodeoxyglucose (FDG)-positron emission tomography/CT (PET/CT). The tumor size decreased significantly to 12 mm, a 65.7% reduction, after combined therapy with eight doses of WT1/MUC1-DC and erlotinib for 237 days based on CT and FDG uptake by PET/CT. After 321 days of combination therapy, no tumor was detected based on CT. Validation of the tumor’s disappearance persisted for at least 587 days after treatment initiation, without any indication of recurrence or metastasis. Red circles show lung cancer

It is essential to evaluate the antitumor immunity induction initiated by the WT1/MUC1-DC vaccine. In this case, we assessed the induction of antitumor immunity by erythema size at vaccinated sites and neutrophil-to-lymphocyte ratio (NLR). The erythema size was gauged at 48 hours following vaccination, as it is linked to immune response development [5, 9, 10]. The vaccination sites demonstrated an erythema diameter of 30 mm after the first vaccination, which persisted above 20 mm throughout the vaccination period in addition to the NLR, which is considered by Jiang and Huai as a prognostic factor and potentially predictive biomarker in immunotherapy [12, 13]. However, the NLR is not universally accepted or used as such, although it is supported by data obtained in several (but not all) tumor types. In this patient, the NLR decreased from 4.6 (baseline) to 1.6 (after 13 vaccinations). Remarkably, the tumor size decreased significantly to 12 mm, a 65.7% reduction, after combined therapy with eight doses of WT1/MUC1-DC and erlotinib for 237 days (Fig. 3). The patient requested a dosage reduction of erlotinib to 100 mg/day based on the observed positive therapeutic response. Moreover, no lung cancer was detected on CT scans at 321 days after treatment (Fig. 3). At that point, ten vaccinations had been given. Validation of the tumor’s disappearance persisted for at least 587 days after treatment initiation, without any indication of recurrence or metastasis (between December 2017 and November 2019; Fig. 3). Additionally, the vaccine dose was increased to 2 × 10^7^ cells per dose (from eight to ten doses), whereas the erlotinib dose was reduced. Later, the vaccine dose was reduced again to approximately 1 × 10^7^ cells after 11–15 doses. In addition, no adverse events were documented during the administration of 15 doses of WT1/MUC1-DCs throughout the treatment period. The patient continued to take erlotinib due to tolerable side effects. At the patient’s request, all 15 vaccine doses were administered following the completion of 699 days of treatment.

## Discussion

This is a meaningful report of a patient with NSCLC who had achieved remission after continued treatment with the WT1/MUC1-DC vaccine and erlotinib. In DC vaccine settings, WT1 killer peptides restricted to HLA-A*24:02, HLA-A*02:01, and HLA-A*02:06 are usually available in Japan. However, the patient’s HLA types were HLA-A*(31:01/33:03), for which a vaccine was not available. Therefore, WT1-specific helper peptides, which can be used for all MHC class II types, and MUC1 long peptides were used in this case. The WT1/MUC1-DC vaccine was characterized by flow cytometry, and the phenotype showed high levels of migration marker (CCR7), MHC molecules (HLA-ABC and DR), costimulatory molecules (CD80 and CD86), and maturation marker (CD83), all of which are associated with migration of WT1/MUC1-DC cells into T-cell areas of lymph nodes, initiation of antigen presentation, and induction of antigen-specific T-cell responses [4]. Importantly, the WT1 helper peptide can significantly contribute to tumor eradication not only through helper CD4^+^ T cells but also WT1-specific CD8^+^ CTLs in antitumor immunity [14]. The MUC1 long peptide can also be used for all MHC class I types to induce MUC1-specific immune responses [9]. Therefore, WT1- and MUC1-specific CTLs might have been induced by WT1/MUC1-DC vaccination in this patient.

The WT1 and/or MUC1-specific immune response can be evaluated by a tetramer assay in clinical settings; however, adequate HLA-A*31:01/33:03 and HLA-DRB1/DPB1 tetramers were not available. However, WT1- and MUC-specific immune responses may be also induced by the WT1/MUC1-DC vaccine in this patient, as noted in other clinical trials [5, 9, 10]. In fact, the erythema at the vaccination site was 30 mm even after the first vaccination and continued for more than 20 mm during long-term vaccination periods. A previous report indicated that erythema at the vaccination site (30 mm in longitudinal diameter or more) is associated with induction of antitumor immunity and clinical benefits from the WT1/MUC1-DC vaccine [10]. This patient had a 30 mm diameter erythema, suggesting induction of antitumor immunity by the vaccine. We also assessed the NLR in this patient. A previous study showed that an elevated blood NLR is associated with shorter PFS and OS in patients with NSCLC treated with PD-1/PD-L1 inhibitors [12]. In this case, a decrease in NLR from baseline during treatment may correlate with long-term survival. However, antitumor immunity is specific and complex, and its assessment is still a challenge even after several years of experience with several immune-related therapies. The vaccination-site erythema size and NLR only support the general (nonspecific) immunogenicity of a vaccine. A rebiopsy of one or more tumor sites would have been of much use to assess the actual antitumor immune response (for example, tumor infiltration lymphocytes), but tumor tissue was not available after treatment in this case.

It has been documented that patients with EGFR-mutated NSCLC respond well to first-line EGFR tyrosine kinase inhibitors [15]. The phase III randomized trials OPTIMAL [16] and EURTAC [17] reported that PFS (13.1 months and 9.7 months, respectively) was significantly better than chemotherapy (4.6 months and 5.2 months, respectively) in patients with EGFR-mutated NSCLC receiving first-line erlotinib alone. Interestingly, following treatment with erlotinib in combination with the WT1/MUC1-DC vaccine, the tumor was undetectable by CT analysis for at least 587 days after treatment in a patient with lung cancer with EGFR-mutated adenocarcinoma. Taken together, these findings suggest that it might be reasonable to use the WT1/MUC1-DC vaccine for such patients.

## Conclusion

The WT1/MUC1-DC vaccine was well tolerated and no treatment-related adverse events were observed. Combined treatment with standard anticancer therapy and the WT1/MUC1-DC vaccine may induce antitumor immune responses during vaccination and appears to provide some clinical benefit to patients with cancer. A large prospective study is warranted to evaluate the clinical benefit of this treatment modality.

## Data Availability

The datasets during the current study are available from the corresponding author on reasonable request.

## Abbreviations

DCs: Dendritic cells
WT1: Wilms’ tumor 1
MUC1: Mucin 1
WT1/MUC1-DC: Dendritic cells pulsed with Wilms' tumor 1 and mucin 1
CT: Computed tomography
EGFR: Epidermal growth factor receptor,
ALK: Anaplastic lymphoma kinase
PD-L1: Programmed death ligand 1
FDG: Fluorodeoxyglucose
PET/CT: Positron emission tomography/computed tomography
NSCLC: Non-small cell lung cancer
OS: Overall survival
PFS: Progression-free survival
TAAs: Tumor-associated antigens
CEA: Carcinoembryonic antigen
CA19-9: Carbohydrate antigen 19–9
CA125: Cancer antigen 125
CA15-3: Cancer antigen 15–3
PS: Performance status
MHC: Major histocompatibility
CTLs: Cytotoxic T lymphocytes
PBMC: Peripheral blood mononuclear cells
HLA: Human leukocyte antigen
NLR: Neutrophil-to-lymphocyte ratio
